# The *Escherichia coli* O157:H7 bovine rumen fluid proteome reflects adaptive bacterial responses

**DOI:** 10.1186/1471-2180-14-48

**Published:** 2014-02-21

**Authors:** Indira T Kudva, Thaddeus B Stanton, John D Lippolis

**Affiliations:** 1 Food Safety and Enteric Pathogens Research Unit, National Animal Disease Center, Agricultural Research Service, U.S. Department of Agriculture, Ames, Iowa 50010, USA; 2 Ruminant Diseases and Immunology Research Unit, National Animal Disease Center, Agricultural Research Service, U.S. Department of Agriculture, Ames, Iowa 50010, USA

**Keywords:** O157, Rumen, LC-MS/MS, iTRAQ, Proteome, Growth-patterns

## Abstract

**Background:**

To obtain insights into *Escherichia coli* O157:H7 (O157) survival mechanisms in the bovine rumen, we defined the growth characteristics and proteome of O157 cultured in rumen fluid (RF; pH 6.0-7.2 and low volatile fatty acid content) obtained from rumen-fistulated cattle fed low protein content “maintenance diet” under diverse *in vitro* conditions.

**Results:**

Bottom-up proteomics (LC-MS/MS) of whole cell-lysates of O157 cultured under anaerobic conditions in filter-sterilized RF (fRF; devoid of normal ruminal microbiota) and nutrient-depleted and filtered RF (dRF) resulted in an anaerobic O157 fRF-and dRF-proteome comprising 35 proteins functionally associated with cell structure, motility, transport, metabolism and regulation, but interestingly, not with O157 virulence. Shotgun proteomics-based analysis using isobaric tags for relative and absolute quantitation used to further study differential protein expression in unfiltered RF (uRF; RF containing normal rumen microbial flora) complemented these results.

**Conclusions:**

Our results indicate that in the rumen, the first anatomical compartment encountered by this human pathogen within the cattle gastrointestinal tract (GIT), O157 initiates a program of specific gene expression that enables it to adapt to the *in vivo* environment, and successfully transit to its colonization sites in the bovine GIT. Further experiments *in vitro* using uRF from animals fed different diets and with additional O157 strains, and *in vivo* using rumen-fistulated cattle will provide a comprehensive understanding of the adaptive mechanisms involved, and help direct evolution of novel modalities for blocking O157 infection of cattle.

## Background

*Escherichia coli* O157 (O157) have been implicated in several human outbreaks since their being established as foodborne pathogens in 1982; an estimated 63,153 illnesses, 2,138 hospitalizations and 20 deaths occur annually in the United States [1-4]. Human disease ranges from self-limiting watery diarrhea to debilitating bloody diarrhea that can advance into often fatal, extraintestinal, secondary sequelae in susceptible patients [3,4]. Cattle are the primary reservoirs for O157, with their recto-anal junction (RAJ) serving as the colonization site at which these human foodborne pathogens persist [4,5]. However, the first bovine gastrointestinal tract (GIT) compartment that O157 encounters is the rumen, where the dynamic environment of regurgitating food particles, bio-fermentation, changing pH, and production of varying amounts of volatile fatty acids (VFA) poses challenges for O157 survival [6-8]. Studies thus far, have concentrated on the recovery of O157 from the rumen, the *in vitro* O157 growth dynamics in modified rumen fluid or media with additives to mimic the rumen environment, expression of select O157 genes under controlled pH and VFA conditions, dietary effects on bacterial survival, and effects of select flora/metabolite on the growth/survival of O157 in the rumen or rumen fluid [6-11]. Despite this, however, a comprehensive study of the mechanisms used by O157 to survive the rumen environment is yet to be undertaken. Hence, as an initial step, we determined the repertoire of O157 proteins (proteome) as expressed *in vitro* in harvested, rumen fluid (RF). We included RF of varying compositions (with and without normal flora, or depleted of nutrients essential for bacterial growth), with no additives, and used diverse culture conditions, to identify bacterial factors that may enable O157 adaptation to the rumen.

## Methods

### Bacterial strain, inoculum preparation and animals

Wild-type O157 strain 86–24 (Shiga toxin (Stx) 1-negative, Stx 2-positive; motile; clinical isolate) was used in this study [12]. Overnight culture of O157 in Luria-Bertani (LB) broth, grown at 39°C with aeration was used to prepare log-phase sub-cultures of the same in 50 ml LB broth, under the same growth conditions. Bacteria harvested from the log-phase cultures at an OD_600_ 0.5-0.6, washed and re-suspended in sterile 0.9% saline, were used to inoculate various rumen fluid (RF) or LB aliquots as described under ‘Culture conditions and processing for proteomics’. All O157 cultures were confirmed serologically using latex agglutination kits (Remel Inc., Lenexa, KS). Two rumen-fistulated Holstein cows, routinely used as rumen fluid ‘donors’ at the National Animal Disease Center (NADC, Ames, IA) with approval from the NADC-Animal Care and Use Committee, were used in this study. Both animals, approximately 1 year of age, were fed the NADC Maintenance Diet (corn silage, grass hay, 520 pellets, protein supplements) at 25% fiber and 10% protein, with ad-lib access to water through out.

### Unfiltered (uRF), Filtered (fRF), and Depleted RF (dRF)

Rumen fluid samples collected from the two animals (Samples A and B; Tables 1 and 2), on separate days, were used to prepare the RF-preparations for each experiment set (Experiment I and II). Two liters of RF was collected 2–3 hr post-feeding to allow for rumination to occur, at each sampling time [10,13]. RF was strained through cheesecloth to remove large feed particles, and poured into collection flasks; pH was recorded on site and an aliquot frozen at –80°C for volatile fatty acid (VFA) analysis. Approximately 500 ml of the strained RF was stored as the unfiltered RF (uRF) at 4°C.

**Table 1:** Biochemical characteristics of rumen fluid used to analyze growth patterns of O157 strain 86–24 in Experiment I

**Sample analysis**	**Depleted rumen fluid**		**Filtered rumen fluid**	
	**Sample A**	**Sample B**	**Sample A**	**Sample B**
pH^1^	7.9	7.6	7.6	7.7
Volatile Fatty Acids (μm/ml; VFA)				
Total VFA	324	207	211	157
Acetic acid	201	142	144	112
	(62%)^2^	(69%)	(68%)	(71%)
Propionic acid	41	28	31	23
	(13%)	(14%)	(15%)	(15%)
Butyric acid	43	20	16	10
	(13%)	(10%)	(8%)	(6%)

^1^pH, post-depletion and/or post-filtration of the depleted and filtered rumen fluid samples, respectively.

^2^Percent individual volatile fatty acid of the total is shown in parenthesis.

**Table 2:** Biochemical characteristics of rumen fluid used to analyze growth patterns of O157 strain 86–24 in Experiment II

**Sample analysis**	**Depleted rumen fluid**		**Filtered rumen fluid**		**Unfiltered rumen fluid**	
	**Sample A**	**Sample B**	**Sample A**	**Sample B**	**Sample A**	**Sample B**
pH^1^	7.6	7.4	7.7	7.2	6.4	6.7
Volatile Fatty Acids (μm/ml; VFA)						
Total	203	205	144	153	210	165
Acetic acid	139	140	103	110	141	104
	(68%)^2^	(68%)	(72%)	(72%)	(67%)	(63%)
Propionic acid	28	28	21	23	32	30
	(14%)	(14%)	(13%)	(15%)	(15%)	(18%)
Butyric acid	19	19	9	10	20	17
	(9%)	(9%)	(6%)	(7%)	(10%)	(10%)

^1^pH, post-depletion and/or post-filtration of the depleted and filtered rumen fluid samples, respectively.

^2^Percent individual volatile fatty acid of the total is shown in parenthesis.

One half of the remaining strained RF was processed as follows to generate filtered RF (fRF). The strained RF was centrifuged at 27,000× g for 30 mins at 18°C, at least 3 times, to remove particulate matter and pressure filtered using a 0.5 μ pre-filter and a 0.2 μ filter in tandem (Pall Corporation, Port Washington, NY). The fRF was collected into sterile bottles and stored at 4°C after recording the pH and freezing an aliquot for VFA analysis.

To prepare dRF, the other half of the remaining strained RF was first subjected to depletion, a process that involves exhaustion of residual nutrients in the RF by exploiting metabolic activities of the resident microflora, prior to the centrifugation-filtration steps. Specifically, the depletion process was initiated by adjusting the strained RF pH to 6.8-7.0, and incubating it under anaerobic conditions, at 39°C for four days. The strained RF was held in flasks fitted with stoppers bearing valves to release the fermentation gases throughout the incubation, following which the depleted RF was centrifuged and filtered as described above. This depletion protocol was adapted from previously described methods with no extraneous substrates added to the RF prior to depletion [11,14]. The pH of the resultant filter-sterilized dRF was recorded and aliquots set aside for VFA analysis prior to storage at 4°C in sterile bottles.

### pH and volatile fatty acids (VFA) analysis

Initial rumen fluid pH measurements were taken during collection by using a portable pH meter (Thermo Fisher Scientific Inc., Waltham, MA) [8,11]. Subsequently, the pH meter or pH paper was used (pH range 5.0–8.0; Micro Essential Laboratory Inc., Brooklyn, NY), to record pH of the processed RF and media. VFA concentrations in rumen fluid and its preparations were determined by capillary gas chromatography of their butyl esters, as described previously [15,16], on an Agilent 6890 N gas chromatograph (Agilent Technologies, Inc., Santa Clara, CA).

### Culture conditions, and processing for proteomics

RF preparations from Samples A and B were analyzed separately per experiment set, and each analysis in turn was conducted in duplicate. In Experiment I, 5 ml LB, dRF, or fRF media were aliquoted separately into 85, 16 × 150 mm tubes. Of these, five tubes per media were used as uninoculated controls. The remaining 80 tubes were inoculated with O157. To create anaerobic culture conditions, half of these tubes were transferred into the anaerobic Coy Chamber for 72 hrs, sealed and inoculated within the chamber and then removed. The log-phase O157 culture, re-suspended in 0.9% saline was inoculated to a starting OD_600_ 0.05-0.06, into all the 80 tubes, which were then incubated at 39°C with shaking, along with the uninoculated control tubes. O157 was grown to an OD_600_ of 0.8-1.0, before harvesting cells from each tube by centrifugation at 7,000 rpm, 15 min at 4°C. Bacterial cells from like media, whether derived from RF-samples A or B, were pooled together and washed three times with an equal volume of ice-cold sterile phosphate buffered saline (PBS; pH 7.4), and processed to obtain cell lysate and pellet fractions for bottom-up proteomic analysis [17].

In Experiment II, uRF was included to the media (LB, dRF, fRF) being evaluated and aliquoted as described above. However, the O157 inoculum diluted in saline to the starting OD_600_ 0.05-0.06 was placed in sterile dialysis tubing (Spectra/Por Type F, PVDF: 80,000 kDa cut off; Serva Electrophoresis, Heidelberg, Germany) and suspended within the uRF containing tubes [18]. This was to ease the recovery of O157 from the complex uRF milieu and the colony counts recovered from the tubings matched those obtained by magnetic recovery of O157 from directly inoculated uRF (data not shown). O157-innoculated LB, dRF, fRF, and uRF were incubated for 48 h, anaerobically, before harvesting cells and processing for proteomic analysis [17] using iTRAQ. For this experiment, bacterial cells from like media were pooled together but kept separate between preparations derived from RF-samples A and B. The culture conditions used in Experiment II correlated with ruminal conditions and feed turnover rates [19-21]. In both experiments, OD_600_ of each tube was recorded relative to uninoculated control tubes, centrifuged at 10,000 rpm for 10 min to remove any sediments or particulate matter which could interfere with the spectrophotometer reading. In addition, pH, and colony counts (on LB agar) were determined from the five uninoculated and ten inoculated tubes at different time points, for comparison.

### Bottom-up proteomics using liquid chromatography tandem mass spectrometry (LC-MS/MS)

This proteomic analysis was done at the Proteomics Division, ICBR, University of Florida, Gainesville, Florida. O157 cell pellet and lysate fractions from Experiment I (LB, dRF, fRF) were concentrated using spin filters (MW cutoff 5000 Daltons), and digested with trypsin prior to tandem mass spectrometry (MS/MS) as described previously [17]. The enzymatically-digested samples were injected onto a capillary trap (LC Packings PepMap) and desalted for 5 min with a flow rate of 3 μl/min of 0.1% v/v acetic acid. The samples were loaded onto an LC Packing® C18 Pep Map nanoflow HPLC column. The elution gradient of the HPLC column started at 3% solvent B, 97% solvent A and finished at 60% solvent B, 40% solvent A for 95 min for protein identification.

Solvent A consisted of 0.1% v/v acetic acid, 3% v/v acetonitrile (ACN), and 96.9% v/v H_2_O. Solvent B consisted of 0.1% v/v acetic acid, 96.9% v/v ACN, and 3% v/v H_2_O. LC-MS/MS analysis was carried out on a hybrid quadrupole-TOF mass spectrometer (QSTAR elite, Applied Biosystems, Framingham, MA). The focusing potential and ion spray voltage was set to 225 V and 2400 V, respectively. The information-dependent acquisition (IDA) mode of operation was employed in which a survey scan from m/z 400–1800 was acquired followed by collision-induced dissociation (CID) of the four most intense ions. Survey and MS/MS spectra for each IDA cycle were accumulated for 1 and 3 s, respectively.

Tandem mass spectra were extracted by ABI Analyst version 2.0. All MS/MS samples were analyzed using Mascot (Matrix Science, London, UK; version 2.2.2). Mascot was set up to search NCBI with taxonomy Bacteria database assuming the digestion enzyme trypsin. Mascot was searched with a fragment ion mass tolerance of 0.50 Da and a parent ion tolerance of 0.50 Da. Iodoacetamide derivative of Cys, deamidation of Asn and Gln, oxidation of Met, were specified in Mascot as variable modifications. Scaffold (version Scaffold-03-3-2, Proteome Software Inc., Portland, OR) was used to validate MS/MS based peptide and protein identifications. Peptide identifications were accepted if they could be established at greater than 95.0% probability as specified by the Peptide Prophet algorithm [22]. Protein identifications were accepted if they could be established at greater than 99.0% probability and contained at least 2 identified unique peptides. Proteins with single peptide hits were included if they exhibited high confidence based on low false discovery rates [23]. Relative protein abundance was estimated using the normailized total spectral counts [24]. Protein probabilities were assigned using the Protein Prophet algorithm [25]. Proteins that contained similar peptides and could not be differentiated based on MS/MS analysis alone were grouped to satisfy the principles of parsimony.

### Quantitative proteomics using isobaric tags for relative and absolute quantification (iTRAQ)

O157 cell pellet and lysate fractions from Experiment II (LB, dRF, fRF, uRF; cultured 48 h, anaerobically) were analyzed by iTRAQ. Samples were processed, trypsin digested, and labeled with various iTRAQ reagents as described earlier [26], in accordance with the manufacture’s instructions for the iTRAQ 4-plex kit (Amine-Modifying Labeling Reagents for Multiplexed Relative and Absolute Protein Quantitation, Applied Biosystems, Foster City CA). Labeled peptides were combined, dried in one tube, and held at -80°C until use. A modification of the previously used protocol was used to analyze these labeled peptides that were resuspended in mobile phase A (72 mM triethlyamine in H2O, pH 10 with acetic acid) at a concentration of 200 μg/μl and incubated for 1 hour in a sonic-water bath at RT. 100 μg of sample was injected into a Waters 1525 μ Binary HPLC (Waters Corporation, Milford, MA) with a Waters XBridge C18, 3.5um, 1 × 100 mm column in mobile phase A and ran isocratically for 6 minutes. The gradient consisted of, 0-20% mobile phase B (72 mM triethlyamine in ACN, 52 mM acetic acid), over 34 minutes; 20-40% over 20 minutes; and finally 40-100% over 2 minutes, at a flow rate of 100 μl/minute throughout the entire gradient [27]. Two-minute fractions were collected, dried in a vacuum centrifuge, and resuspended in nano-HPLC buffer A (95% H_2_O: 5% ACN and 0.1% formic acid). Based on previous experience we combined, 3 fractions before and after, the fractions that contained the majority of the eluted peptides.

Fractions from the first dimension chromatography were injected on a second dimension of chromatography using a Proxeon Easy-nLC (Thermo Fisher Scientific, West Palm Beach, FL) connected to the mass spectrometer. The second dimension chromatography used a trapping column (Proxeon Easy-Column, 2 cm, ID 100 μm, 5um, 120A, C18) and an analytical column (Proxeon Easy-Column, 10 cm, ID 75 μm, 3 μm, 120A, C18). The gradient using a mobile phase A (95% H2O: 5% acetonitrile and 0.1% formic acid) and mobile phase B (5% H2O: 95% acetonitrile and 0.1% formic acid). The gradient was, 0% B for 3 minutes, 0%-8% B from 3–5 minutes, 8-18% B from 5–85 minutes, 18-30% B from 85–100 minutes, 30-90% B from 100–105 minutes, and held at 90% B from 105–120 minutes at continuous flow rate throughout the gradient of 300 nl/min. The analytical column was connected to a PicoTip Emitter (New Objectives, Woburn, MA; FS360-75-15-N-20) and together attached to a LTQ OrbiTrap Velos Pro (Thermo Fisher Scientific, West Palm Beach, FL) mass spectrometer using the Proxeon Nanospray Flex Ion Source. The capillary temperature was set at 275°C and spray voltage was 2.9 kV. The mass spectrometer was used in a data dependent method. In MS mode, the instrument was set to scan 300–2000 m/z with a resolution of 30,000 FWHM. A minimal signal of 20,000 could trigger tandem MS and 10 consecutive MS/MS were possible. High-energy collision-induced dissociation (HCD) was used to resolve the iTRAQ reporter ions, 113–117. The normalized collision energy was set to 35 and repeat mass exclusion was set to 120 seconds.

Tandem mass spectra were extracted and charge state deconvoluted by Proteome Discoverer version 1.4. Charge state deconvolution and deisotoping was not performed. All MS/MS samples were analyzed using Mascot, Sequest (XCorr Only; Thermo Fisher Scientific, San Jose, CA, USA; version 1.3.0.339) and X! Tandem (GPM.org; version CYCLONE (2010.12.01.1)) assuming digestion with trypsin. A custom *E. coli* database was generated by combining the fasta files from uniprot.org from the following *E. coli* strains: 12009/EHEC, 2009EL-2050, 2009EL-2071, 2011C-3493, 11128/EHEC, O157:H7, EC4115/EHEC, TW14359/EHEC, and 11368/EHEC. This *E. coli* fasta file consists of 47,819 entries and was generated in May 2013. Mascot, Sequest (XCorr Only) and X! Tandem were searched with a fragment ion mass tolerance of 0.100 Da and a parent ion tolerance of 10.0 PPM; carbamidomethyl of cysteine and iTRAQ4plex of lysine and the n-terminus were specified as fixed modifications while deamidation of asparagine and glutamine, oxidation of methionine and iTRAQ4plex of tyrosine were specified as variable modifications. Scaffold (version Scaffold_4.0.6) was used to validate MS/MS based peptide and protein identifications, as described above for *‘Bottom-up Proteomics’*. The O157-proteome as expressed in LB was used as the reference against which all the other O157-proteomes were compared. Two biological replicate samples (Sample A and B), corresponding to the duplicate experiments described under ‘*Culture conditions, and processing for proteomics’* above, were analyzed separately. In addition, each sample was analyzed twice (Run A and Run B; technical replicates) to cover the entire spectra of proteins in these samples. Only proteins that were consistently identified were selected for analysis.

### Statistics and bioinformatics

The Student t-Test (two-tailed) was used to evaluate differences between the means of the O157 optical densities and viable counts recovered from the different cultures and a values of *p* < 0.05 was considered significant. Putative functions were determined by querying the Conserved Domain Database (CDD) at http://www.ncbi.nlm.nih.gov/Structure/cdd/wrpsb.cgi, and associated metabolic pathways were determined using the KEGG pathway database at http://www.genome.jp/kegg/pathway.html. Cellular and sub-cellular locations of proteins were determined as described previously [17].

## Results

### pH and VFA content

The pH and VFA concentrations were comparable amongst all rumen fluid samples, indicating consistency in maintenance diet being fed and the ruminal chemistry between the two animals enrolled in the study (Tables 1 and 2). The pH of the uRF ranged from 6.4-6.7 at collection [28-31] but attained a more neutral pH after filtering, as seen with dRF (pH 7.4–7.9) and fRF (pH, 7.2–7.7) in both experiments (Tables 1 and 2). Concentrations of three VFAs, acetate, propionate and butyrate, were closely analyzed as these vary the most with changes in the forage versus starch compositions of the feed, and are of relevance to both host and bacterial growth. Consistent with the 25% forage and 10% protein diet that these cattle were being fed, the RF comprised a higher percentage of acetate [28-31]. Acetate ranged from 72-62%, compared to the 13-18% propionate and 6-13% butyrate concentrations across the uRF, dRF and fRF samples in both experiments, irrespective of procedures used to prepare dRF and fRF (Tables 1 and 2). LB broth (pH 7.0-7.2) did not contain added VFAs.

### O157 growth characteristics

Log phase O157 cultures, set up for the two experiments, were at 0.5-0.6 OD_600_, respectively, with viable counts around 1 × 10^8^ cfu/ml. Hence, when each medium was inoculated to a starting 0.05-0.06 OD_600_, the corresponding O157 counts were at ~1-5 × 10^7^ cfu/ml. In both experiments, O157 grew to an OD_600_ of 1.0 within 2 h in LB media, aerobically and anaerobically as anticipated, with an increase in viable count to 4 × 10^8^ cfu/ml and the final culture pH at 6.0-6.2. However, significant differences were observed between aerobic and anaerobic growth patterns of O157 when cultured in dRF, fRF and uRF preparations.

**Figure 1:**
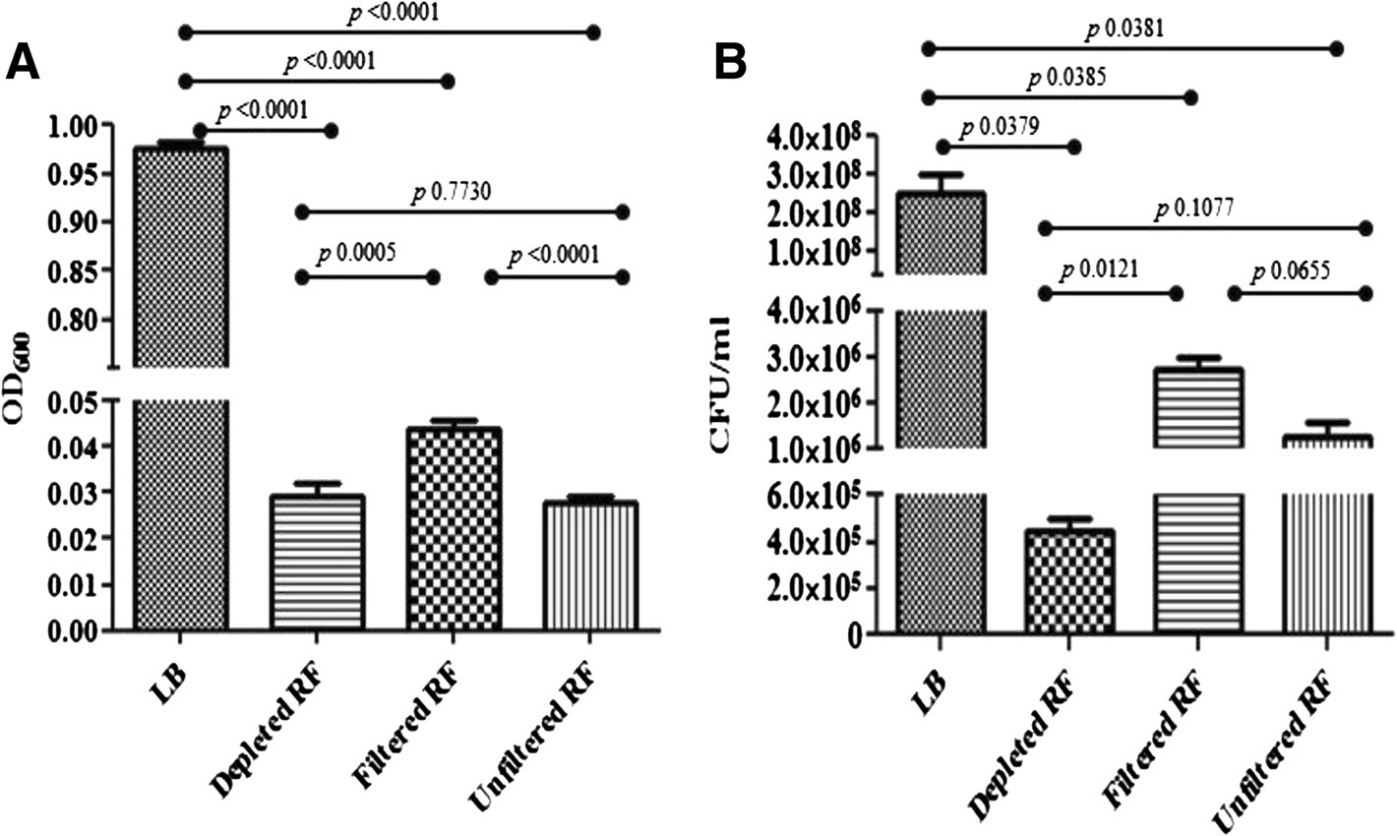
**Growth characteristics of O157 in Experiment II, following anaerobic incubation for 48 h, in LB and RF-preparations.** Optical densities (OD_600_) and viable counts (colony forming units [cfu]/ml), with the standard error of means, are shown in graph **A** and **B**, respectively. The *p* values shown on the graphs were calculated using the Student t-Test (significant, *p* < 0.05).

In Experiment I, O157 cultured in dRF and fRF achieved an average OD_600_ of 0.6-1.0 in 48 h aerobically, but remained at a low OD_600_ of ≤0.2 anaerobically, even after 14 days of incubation. Irrespective of the ODs, viable O157 was recovered from all cultures, but the viable counts at 10^6^ (dRF)-2 × 10^7^ (fRF) cfu/ml aerobically, and at 10^5^ (dRF)-2 × 10^5^ (fRF) cfu/ml anaerobically (data not shown) appeared to be static or decreasing. The pH for dRF and fRF cultures at the end of incubation was around 7.7 (aerobic)–7.3 (anaerobic). Similar O157 growth results were observed upon anaerobic culture for 48 h in dRF, fRF and uRF, in Experiment II (Figure 1), with the pH for uRF cultures being 6.8 at end of incubation. This was despite these media being prepared with RF from a separate animal and a shorter anaerobic incubation period than in the first experiment, thereby verifying the observations made initially. Here, the cultures reached an average OD_600_ of 0.97 (LB), ~0.03 (dRF), ~0.04 (fRF) and ~0.03 (uRF) in 48 h, with O157 viable counts of 2 × 10^8^ cfu/ml (LB), 4 × 10^5^ cfu/ml (dRF), 3 × 10^6^ cfu/ml (fRF) and 1 × 10^6^ cfu/ml (uRF), respectively.

Significant differences were observed among the optical densities and viable counts of LB cultures versus RF-preparation cultures, under all growth conditions. However, differences between the RF-preparations were not always significant (Figure 1). For instance, in Experiment II, the *p* values for the O157 viable counts were: LB: dRF, *p =* 0.0379; LB: fRF, *p =* 0.0385; LB: uRF, *p =* 0.0381, dRF: fRF, *p =* 0.0121 and fRF: uRF, *p =* 0.0655; dRF: uRF, *p =* 0.1077.

### Proteomics analysis

#### (i) Bottom-up

**Figure 2:**
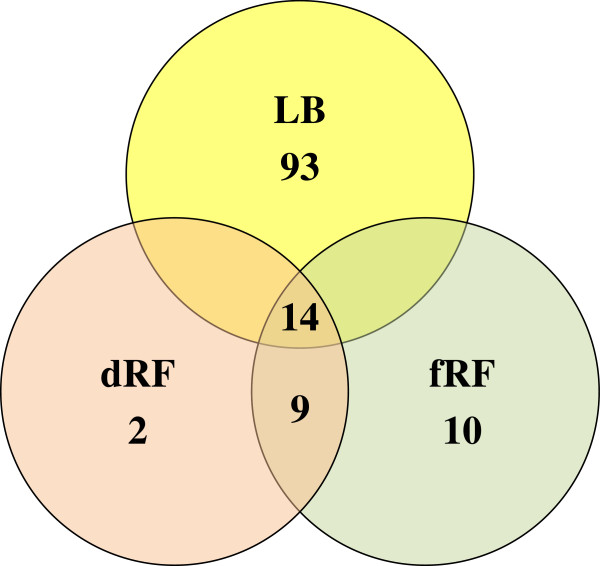
**Distribution of 128 anaerobically expressed O157 proteins, identified using bottom-up proteomics, amongst the media tested.** LB, Luria-Bertani broth; dRF, depleted and filtered rumen fluid; fRF, filtered rumen fluid.

LC-MS/MS analysis of the O157 cell pellet and lysate fractions generated in Experiment I provided insights into the proteins being expressed by O157 in different media, under different growth conditions and at extended incubation time points. A total of 585 protein (2284 spectra) hits were identified by setting minimum characteristics for the identification confidence. However, of these only 218 O157 proteins matched a higher threshold cut off, with 90% protein-80% peptide probability in the Scaffold Viewer, and hence, were selected for analysis. The 218 O157 proteins were differentially expressed: 90 only under aerobic conditions, 37 only under anaerobic conditions and 91 under both conditions (data not shown), accounting for fewer proteins under anaerobic conditions. Interestingly, none of the O157 proteins expressed aerobically or anaerobically in either media were associated with direct virulence (e.g., the Locus of Enterocyte Effacement [LEE]-encoded proteins or Shiga toxins) but were primarily associated with sequences homologous to other *E. coli* genomes (Backbone) (Additional file 1: Table S1). Considering that the rumen is an anaerobic microbiome, the 128/218 O157 proteins expressed anaerobically were examined in greater detail. These proteins were either unique to growth in LB (93/128), dRF (2/128), fRF (10/128) or, expressed in more than one media (14/128 in LB/dRF/fRF, 9/128 in dRF/fRF) (Figure 2). Specifically, the 35 proteins expressed anaerobically in fRF and dRF (unique and shared combined), were functionally associated with the osmotic adaptation pathway (OsmE), anaerobic respiration and oxidative stress pathway (YggE, MoaB, DmsB, FdoH), heat stress response (HchA), carbon starvation response (Slp), energy metabolism and biosynthetic pathways (glycolytic/gluconeogenesis pathway, amino acid biosynthesis: AldoC, Crr, AnsB, PykF, Eno, GpmA, GadpH, CysK, Ttc, AhpC, YhcB), chaperones (DnaK, GroEL, HchA), transport (LamB, ManX, FadL, RbsB), outer membrane proteins/porins/channel (OmpC, TolC, YdeN, Slp, OmpA), tellurite resistance (TerD), lysozyme inhibitor (Ivy), chemotaxis (GgbP), and motility (FliC) (Table 3; Additional file 1: Table S1).

**Table 3:** O157-proteome expressed under anaerobic conditions in dRF and fRF in Experiment I

**Protein/Function/Pathway; Name**	**Accession Number**	**Molecular Weight (kDa)**^**1**^	**Number of Peptides (Relative Abundance)**^**2**^	
			**dRF**	**fRF**
2,3-bisphosphoglycerate-dependent phosphoglycerate mutase; **GpmA**	gi|157155502	29 kDa	1 (6)	1 (4)
3-isopropylmalate dehydrogenase/Amino acid Biosynthesis; **TtC**^3^	gi|170684236	40 kDa	1 (6)	1 (4)
Alkyl hydroperoxide reductase protein C/Energy; **AhpC**	gi|15800320	21 kDa	0	1 (4)
Anaerobic dimethyl sulfoxide reductase/Anaerobic growth/Oxidative Stress; **DmsB**	gi|145756	23 kDa	1 (6)	1 (4)
Chain A, Crystal Structure Of The Long-Chain Fatty Acid Transporter/Transport; **Fadl**	gi|203282230	47 kDa	1 (6)	1 (4)
Chain A, Crystal Structure Of Ggbp. Glucose-galactose binding protein/Chemotaxis, Transport; **Ggbp**^3^	gi|126030485	33 kDa	0	1 (4)
Chain A, Structure Of Ivy/ Lysozyme inhibitor; **Ivy**	gi|29726212	15 kDa	1 (6)	1 (4)
Chaperone protein, stabilizes proteins under heat stress/Heat Stress Related; **HchA**	gi|15802400	31 kDa	0	1 (4)
Chaperonin, type 1 protein/Protein folding/Transport; **GroEL**^3^	gi|15834378	57 kDa	3 (18)	3 (11)
Cysteine synthase/Amino acid transport and Metabolism; **CysK**	gi|145686	35 kDa	1 (6)	1 (4)
Cytochrome d ubiquinol oxidase subunit III/Oxidative phosphorylation/Energy; **YhCB**^3^	gi|157148804	15 kDa	0	1 (4)
D-ribose transporter subunit B/Transport; **RbsB**	gi|110644091	31 kDa	1 (6)	0
DNA-binding transcriptional activator/Osmotically-inducible lipoprotein E; **OsmE**	gi|15802150	12 kDa	2 (18)	2 (7)
DNA-directed RNA polymerase subunit alpha/Transcription; **RpoA**	gi|123444073	37 kDa	0	1 (4)
Flagellin/Flagellar assembly/Motility; **FliC**	gi|15802358	60 kDa	3 (24)	4 (19)
Formate dehydrogenase-O, iron-sulfur subunit, energy metabolism/Anaerobic Respiration, Glyoxylate & Dicarboxylate Metabolism; **FdoH**	gi|15804482	33 kDa	0	1 (4)
Fructose-bisphosphate aldolase/Glycolysis, Gluconeogenesis, Amino acid Biosynthesis; **AldoC**	gi|161984958	38 kDa	3 (24)	4 (19)
Glucose-specific PTS system component, phosphorylation/Transport; **Crr**	gi|15802950	18 kDa	0	1 (4)
Glyceraldehyde 3-Phosphate Dehydrogenase; **GadpH**^3^	gi|1421424	35 kDa	2 (12)	1 (4)
Hypothetical protein CKO_00658/ Uncharacterized; **DedA**^3^	gi|157144929	21 kDa	0	1 (4)
Hypothetical protein EcE24377A_0553/Glyoxylate Utilization; **GlxB**	gi|157157046	29 kDa	1 (6)	0
Hypothetical protein ECP_2911/Oxidative Stress; **YggE**	gi|110643066	25 kDa	0	2 (7)
L-asparaginase II, induced by anaerobiosis/Nitrogen and Amino acid Metabolism; **AnsB**	gi|157157301	37 kDa	0	4 (15)
Maltoporin/Receptor for lambda phage/Transport; **LamB**^3^	gi|110644375	50 kDa	1 (6)	1 (4)
Molecular chaperone/Protein folding/Transport; **DnaK**	gi|157159481	69 kDa	7 (42)	4 (15)
Molybdopterin biosynthesis protein B/Cofactor Biosynthesis/ Oxidative Stress; **MoaB**	gi|15800533	19 kDa	0	1 (4)
Outer membrane channel protein, efflux of hydrophobic molecules/Transport; **TolC**	gi|110643281	54 kDa	0	2 (7)
Outer membrane porin protein C/Tranport of small molecules/Osmotic; **OmpC**^3^	gi|15802768	41 kDa	1 (6)	5 (22)
Outer membrane protein II, porin, receptor, integrity/Membrane Stability; **OmpA**	gi|146983	26 kDa	2 (12)	3 (11)
Outer membrane protein induced after carbon starvation, stationary phase, environmental stress/ Membrane stability; **Slp**	gi|110807343	27 kDa	0	1 (4)
Phosphopyruvate hydratase: enolase/Glycolysis, Gluconeogenesis; **Eno**^3^	gi|15832893	46 kDa	0	1 (4)
PTS system, mannose-specific IIAB component/phosphotransferase/Transport; **ManX**	gi|110641934	35 kDa	0	1 (4)
Putative sulfatase/Inorganic ion transport and metabolism/Transport; **YdeN**^3^	gi|110641672	63 kDa	0	1 (4)
Pyruvate kinase/Glycolysis, Gluconeogenesis, Amino acid Biosynthesis; **PykF**	gi|110805653	59 kDa	0	1 (4)
Tellurium resistance protein/Stress related; **TerD**	gi|135596	20 kDa	0	1 (4)

^1^KDa, Kilodalton.

^2^Relative abundance based on normalized total spectral counts.

^3^Proteins not identified in Experiment II (see Table 4).

#### (ii) iTRAQ

**Figure 3:**
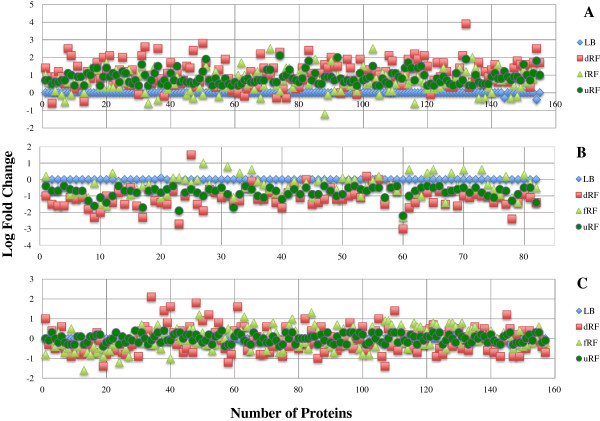
**Log fold changes in the expression of O157 proteins, identified using iTRAQ, in media tested under anaerobic conditions.** The O157-proteome expressed in LB was the reference against which the regulation of O157 proteins in other media was determined. The scatter plots represent O157 proteins expressed in the context of the 155 up-regulated in uRF **(Panel A)**, 82 down-regulated in uRF **(Panel B)** and 157 with no change in expression levels in uRF **(Panel C)**. LB, Luria-Bertani broth; dRF, depleted and filtered rumen fluid; fRF, filtered rumen fluid; uRF, unfiltered rumen fluid.

To more closely examine and quantify O157 protein expression in the bovine rumen, especially in the uRF, the anaerobic O157-proteome expressed in LB, dRF, fRF and uRF after 48 h incubation was compared using iTRAQ, in Experiment II. Data generated in two runs for each biological replicate was condensed to create a single comprehensive file per sample, and the files for the two biological replicate samples compared (Additional file 2: Table S2) to identify unambiguous proteins. Using the anaerobic O157-proteome expressed in LB as the reference, a total of 394 O157 proteins that were either differentially or similarly expressed in dRF, fRF, and uRF were identified (Figure 3, Additional file 2: Table S2). Of the cumulative 35 O157 proteins expressed anaerobically in dRF and fRF, and identified via Bottom-up proteomics, 10 were not identified using iTRAQ in the second experiment (Table 3). Overall, only 134 proteins were common to the results of the two experiments, indicative of incubation-time related differences in the number and type of proteins expressed. Differentially expressed O157 proteins in the iTRAQ dataset distributed as 298/394 in dRF (169, up-regulated, 129, down-regulated), 241/394 in fRF (162, up-regulated, 79, down-regulated) and 237/394 in uRF (155, up-regulated, 82, down-regulated) (Table 4). Interestingly, similar expression patterns were observed between O157 proteins expressed in dRF and uRF; 90% of dRF-differentially regulated and 71% dRF-no change proteins were similarly expressed in uRF. This may have been due to shared growth conditions (nutrient limitation)/signals in these two media. The competing microflora in uRF may have decreased nutrients in that media.

**Table 4:** O157 proteins up-regulated under anaerobic conditions in uRF, in Experiment II

**Protein/Function/Pathway; Name**	**Accession Number**	**Molecular Weight ( kDa)**^**1**^
2,3-bisphosphoglycerate-dependent phosphoglycerate mutase/Glycolysis/Energy; **GpmA**	GPMA_ECO57	29 kDa
3-oxoacyl-[acyl-carrier-protein] reductase/Fatty acid biosynthesis; **FabG**	Q8X8I5_ECO57	26 kDa
3,4-dihydroxy-2-butanone 4-phosphate synthase, Riboflavin (Vitamin B2) biosynthesis/ Cofactor Bisoynthesis; **RibB**	RIBB_ECO57	23 kDa
30S ribosomal protein S6/Protein Translation; **RpsF**	RS6_ECO57	15 kDa
4-hydroxy-tetrahydrodipicolinate reductase, Leucine synthesis/Amino acid Biosynthesis; **DapB**	DAPB_ECO57	29 kDa
50S ribosomal protein L10/Protein Translation; **RplJ**	RL10_ECO57	18 kDa
50S ribosomal protein L18/Protein Translation; **RplR**	RL18_ECO57	13 kDa
Acetyl-coenzyme A carboxylase carboxyl transferase subunit beta/Fatty acid Biosynthesis; **AccD**	ACCD_ECO57	33 kDa
Acriflavine resistance protein A/Multidrug efflux system/Transport; **AcrA**	B5Z3X3_ECO5E	44 kDa
ADP-L-glycero-D-manno-heptose-6-epimerase/LPS core Biosynthesis/Heat induced; **HldD**	HLDD_ECO57	35 kDa
Agmatinase/Polyamine Biosynthesis/Acid Resistance; **SpeB**	SPEB_ECO57 (+1)	34 kDa
AidA-I adhesin-like protein/Adherence; **AidA**	K0AT24_ECO1C	141 kDa
Aminomethyltransferase/Nitrogen, Amino Acid Metabolism; **GcvT**	GCST_ECO57	40 kDa
Argininosuccinate lyase, Arginine synthesis/ Amino Acid Biosynthesis; **ArgH**	ARLY_ECO57	50 kDa
AsmA protein/LPS Biogenesis; **AsmA**	B5YUC4_ECO5E (+5)	69 kDa
Aspartate carbamoyltransferase regulatory chain/Nucleotide Biosynthesis; **PyrI**	PYRI_ECO57	17 kDa
Aspartate--tRNA ligase/Aminoacyl-tRNA Biosynthesis; **AspS**	SYD_ECO57 (+1)	66 kDa
Aspartate-semialdehyde dehydrogenase/Amino acid Biosynthesis; **AsD**	DHAS_ECO57	40 kDa
ATP synthase subunit b/Energy Production; **AtpF**	ATPF_ECO57	17 kDa
ATP-dependent helicase/DNA Replication, Repair; **HrpB**	Q8X904_ECO57 (+1)	91 kDa
ATP-dependent protease subunit/Proteolysis; **HslV**	HSLV_ECO57	19 kDa
Bacterioferritin/Iron storage and Transport; **BfR**	Q8X890_ECO57	18 kDa
Bacteriophage replication gene A protein/Predicted Phage replication; **ECH74115_3058**	B5YUH3_ECO5E	86 kDa
Beta-D-glucoside glucohydrolase, degradation of small carbon compunds/Biosyntheis of secondary metabolites; **BglX**	Q8X668_ECO57	83 kDa
Bifunctional N-acetylglucosamin-1-phosphate-uridyl transferase/Cell Wall Synthesis; **GlmU**	GLMU_ECO57 (+2)	49 kDa
Bifunctional purine biosynthesis protein/Purine Biosynthesis; **PurH**	PUR9_ECO57	57 kDa
Biofilm regulator/Biofilms, Adherence; **BssR**	BSSR_ECO57	15 kDa
Biosynthetic arginine decarboxylase/Polyamine Biosynthesis, Acid Resistance; **SpeA**	SPEA_ECO57	74 kDa
Biotin carboxyl carrier protein of acetyl-CoA carboxylase/Fatty Acid Biosynthesis; **AccB**	BCCP_ECO57 (+8)	17 kDa
Branched-chain-amino-acid aminotransferase/Amino acid Transport and Metabolism; **IlvE**	ILVE_ECO57	34 kDa
Catabolite repression sensor kinase for PhoB alternative sensor for pho regulon/Global Regulaor; **CreC**	Q8XB07_ECO57	52 kDa
Catalase-peroxidase 1/Prevent Cell, DNA damage/Oxidative Stress; **KatG1**	KATG1_ECO57	80 kDa
Cell Division protein; **ZapB**	ZAPB_ECO5E (+8)	9 kDa
Cellulose synthase subunit/Biofilms, Adherence; **BcsC**	C8TYF1_ECO10 (+2)	126 kDa
Chromosome partition protein/Cell Division; **MukB**	MUKB_ECO57 (+2)	170 kDa
Curli production assembly/transport subunit/Biofilms, Adherence; **CsgG**	B5YVQ8_ECO5E (+1)	31 kDa
Cyclic pyranopterin monophosphate synthase accessory protein/Cofactor Biosynthesis; **MoaC**	MOAC_ECO57	17 kDa
Cytidylate kinase/Nucleotide Biosynthesis; **Cmk**	KCY_ECO57	25 kDa
Dihydrolipoyllysine-residue succinyltransferase component of 2-oxoglutarate dehydrogenase		
Complex/Energy Metabolism; **SucB**	ODO2_ECO57	44 kDa
Dihydroorotase/Nucleotide Biosynthesis; **PyrC**	PYRC_ECO57	39 kDa
DNA helicase/DNA Replication, Transcription; **RecB**	Q8X6M9_ECO57	134 kDa
DNA invertase from prophage CP-933H/DNA Replication; **PinH**	Q8X7L1_ECO57	22 kDa
DNA topoisomerase IV subunit B/DNA Replication; **ParE**	Q8XBQ4_ECO57	70 kDa
DNA-directed RNA polymerase subunit alpha/DNA Transcription; **RpoA**	RPOA_ECO57	37 kDa
DNA-directed RNA polymerase subunit beta/DNA Transcription; **RpoB**	RPOB_ECO57	151 kDa
DNA-directed RNA polymerase subunit omega/DNA Transcription; **RpoZ**	RPOZ_ECO57	10 kDa
Elongation factor P-like protein/Translation, Protein synthesis; **YeiP**	EFPL_ECO57	22 kDa
Evolved beta-D-galactosidase alpha subunit/Degradation of small carbon compounds; **EbgA**	Q8XAM9_ECO57	119 kDa
Ferritin-1/Iron Uptake and Storage; **FtnA**	FTNA_ECO57	19 kDa
Fimbrial usher family protein/Chaperone/Transport; **ECH74115_2118**	B5Z1W3_ECO5E (+5)	81 kDa
Formate dehydrogenase-O major subunit/Energy Metabolism, Anaerobic Respiration; **FdoH**	Q7A9A6_ECO57 (+1)	113 kDa
Fructose-bisphosphate aldolase, class 1/Glycolysis, Gluconeogensis; **FbaB**	B5YV44_ECO5E (+1)	38 kDa
Galactose-1-phosphate uridylyltransferase/Galactose Metabolism; **GalT**	Q8X943_ECO57	40 kDa
Glucans biosynthesis protein G/Osmotic Adaptation; **MdoG**	OPGG_ECO5E	59 kDa
Glutamate--tRNA ligase/Amino acyl tRNA synthesis; **GltX**	SYE_ECO57	54 kDa
Glutamine synthetase/Amino acid Biosynthesis; **GlnA**	GLNA_ECO57	52 kDa
Glutathione synthetase/Cofactors, carriers Biosynthesis; **GshB**	GSHB_ECO57	35 kDa
Glycerol kinase/Glycerolipid Metabolism; **GlpK**	GLPK_ECO57	56 kDa
Glycine dehydrogenase [decarboxylating]/Amino acid Metabolism; **GcvP**	GCSP_ECO57	104 kDa
GMP synthase [glutamine-hydrolyzing]/Nucleotide Biosynthesis; **GuaA**	GUAA_ECO57	59 kDa
GTPase-activating protein/Transcriptional Activator; **YihI**	YIHI_ECO57	19 kDa
Guanylate kinase/Nucleotide Biosynthesis; **GmK**	KGUA_ECO57 (+2)	24 kDa
Hemin import ATP-binding protein/Transport; **HmuV**	HMUV_ECO57	29 kDa
Histidine--tRNA ligase/Amino acyl tRNA synthesis; **HisS**	SYH_ECO57	47 kDa
HTH-type transcriptional regulator/Maltooligosaccharide Uptake and Metabolism; **MalT**	MALT_ECO57	103 kDa
Hydrogenase-2 large chain/Energy Metabolism/Anaerobic Respiration; **HybC**	MBHM_ECO57	62 kDa
Hydroxyacylglutathione hydrolase/Pyruvate Metabolism; **GloB**	GLO2_ECO57	28 kDa
Inosine-5′-monophosphate dehydrogenase/Nucleotide Biosynthesis; **GuaB**	B5Z0X7_ECO5E	54 kDa
Iron-sulfur cluster insertion protein/Growth using alternate electron acceptors; **ErpA**	ERPA_ECO57	12 kDa
Long-chain fatty acid transport protein/Transport; **FadL**	FADL_ECO57	49 kDa
Low-affinity putrescine importer/Transport; **PlaP**	PLAP_ECO57	50 kDa
LPS-assembly lipoprotein/LPS Biogenesis; **LptE**	LPTE_ECO57	21 kDa
Macrolide export ATP-binding/permease protein/Transport; **MacB**	MACB_ECO57	71 kDa
Major outer membrane lipoprotein/Membrane Integrity; **Lpp**	LPP_ECO57	8 kDa
Mannitol-1-phosphate 5-dehydrogenase/Fructose, Mannose Metabolism; **MtlD**	MTLD_ECO57	41 kDa
Membrane, TerC family/CBS/transporter associated protein/Transport/Tellurium Resistance; **TerC**	B5YUC1_ECO5E (+6)	59 kDa
Methyl-accepting chemotaxis protein III, ribose and galactose sensor receptor/Chemotaxis; **Trg**	C8U899_ECO10	59 kDa
Minor curlin protein/Biofilms, Adherence; **CsgB**	B5YVR3_ECO5E (+1)	17 kDa
Multiple Resistance and pH adaptation protein, antiporter/Transport; **Mrp**	B5YV63_ECO5E (+1)	40 kDa
Multidrug resistance efflux protein/Transport; **MdtC**	C8TU07_ECO26 (+2)	111 kDa
Multiphosphoryl transfer protein/Fructose, Mannose Metabolism; **FruB**	PTFAH_ECO57	40 kDa
N-acetylglucosamine-6-phosphate deacetylase/LPS Biosynthesis; **NagA**	NAGA_ECO57	41 kDa
NADH-quinone oxidoreductase subunit C/D/Electron transfer/Energy Metabolism; **NuoC**	NUOCD_ECO57 (+2)	69 kDa
NifU-like protein/Energy Production, Conversion; **NifU**	NIFU_ECO57	14 kDa
Non-LEE-encoded type III secreted effector; **EspX7**	C6URC5_ECO5T (+2)	73 kDa
Oligopeptide ABC transporter, periplasmic oligopeptide-binding protein/Transport; **OppA**	B5YYE8_ECO5E	63 kDa
Osmotically-inducible lipoprotein E/Global Regulator; **OsmE**	OSME_ECO57	12 kDa
Outer membrane lipoprotein/Cell Envelope Biogenesis; **SlyB**	SLYB_ECO57	16 kDa
Outer membrane protein, porin, receptor, integrity/Membrane Stability; **OmpA**	B5YT87_ECO5E	38 kDa
Outer membrane protein assembly factor/Membrane Assembly, Antibiotic Resistance; **BamA/YaeT**	BAMA_ECO57	91 kDa
Outer membrane protein assembly factor/Membrane Assembly, Antibiotic Resistance; **BamD/YfiO**	BAMD_ECO57	28 kDa
Outer membrane protein, efflux protein/Transport; **TolC**	B5YR81_ECO5E (+2)	54 kDa
Outer membrane protein W/Outer Membrane Biogenesis; **OmpW/YciD**	Q8XCB6_ECO57	23 kDa
Oxidoreductase subunit/Oxidative Stress; **DmsA_YnfF**	C6UV29_ECO5T (+6)	90 kDa
Penicillin-binding protein activator/Lipid binding/Putative Adhesin; **LpoB/YcfM**	LPOB_ECO57	23 kDa
Peptidase B/Protein Metabolism; **PepB**	PEPB_ECO57	46 kDa
Peptide chain release factor 1/Protein Translation; **PrfA**	RF1_ECO57	41 kDa
Peptidyl-prolyl cis-trans isomerase A/Post-translational Modification; **PpiA**	PPIA_ECO57	20 kDa
Peptidyl-prolyl cis-trans isomerase/Post-translational Modification; **FklB**	B5Z2L4_ECO5E (+2)	22 kDa
Periplasmic binding protein for nickel/Amino acid Transport and Metabolism; **NikA**	Q8X5U3_ECO57 (+1)	59 kDa
Phenylalanine--tRNA ligase alpha subunit/Amino acyl tRNA synthesis; **PheS**	SYFA_ECO57 (+2)	37 kDa
Phenylalanine--tRNA ligase beta subunit/Amino acyl tRNA synthesis; **PheT**	SYFB_ECO57	87 kDa
Poly (A) polymerase I/DNA Transcription; **PcnB**	PCNB_ECO57 (+3)	54 kDa
Proline--tRNA ligase/Amino acyl tRNA synthesis; **ProS**	SYP_ECO57	64 kDa
Protease IgA1, Serine protease/Protection; **EspP**	K0AWD8_ECO1C	146 kDa
Protein elaB/Uncharacterized; **ElaB**	ELAB_ECO57	11 kDa
Protein grpE, prevents aggregation of denatured proteins/ Heat and Hyperosmotic Shock-Related; **GrpE**	C8U980_ECO10 (+2)	22 kDa
Protein translocase subunit/Transport; **SecD**	SECD_ECO57	67 kDa
PTS system, mannose-specific transporter subunit IID/Transport; **ManZ**	B5YQW0_ECO5E (+1)	31 kDa
Putative anaerobic dimethyl sulfoxide reductase chain A; **DmsA_YnfE**	Q7ABM3_ECO57 (+1)	88 kDa
Putative DNA replication factor encoded within cryptic prophage CP-933P/Hypothetical; **Z6069**	Q8XAD9_ECO57	28 kDa
Putative endopeptidase of prophage CP-933X/Hypothetical; **Z1877**	Q8X704_ECO57	12 kDa
Putative carboxypeptidase/Hypothetical; **YagX**	Q8X6I4_ECO57 (+1)	91 kDa
Putative lipoprotein induced during stationary phase/Stress Response; **YbjP**	Q8X6N7_ECO57 (+1)	19 kDa
Putative homeobox protein/Regulator; **YbgS**	Q8X948_ECO57	13 kDa
Putative lipoprotein/Membrane protein; **LppC**	B5YUN1_ECO5E	20 kDa
Putative membrane protein, peptidase/Uncharacterized; **YibP**	Q8XDE2_ECO57	47 kDa
Putative multimodular enzyme/Energy Metabolism; **Z3719**	Q8XBF4_ECO57	82 kDa
Putative pectinesterase, localizes to cellular poles/Membrane protein; **YbhC**	Q8X891_ECO57	46 kDa
Putative replicase/DNA Replication; **Z5187**	Q8XBZ7_ECO57	37 kDa
Pyridoxine 5′-phosphate synthase/Vitamin B6 (Pyridoxine) synthesis; **PdxJ**	PDXJ_ECO57	26 kDa
Pyruvate dehydrogenase (Dihydrolipoyltransacetylase component)/Energy Metabolism; **AceF**	Q8X966_ECO57	66 kDa
Pyruvate oxidase/Degradation of small carbon compunds; **PoxB**	Q8X6L4_ECO57	62 kDa
RNase E/RNA Degradation; **RnE**	Q8X8J5_ECO57	118 kDa
Serine protease/Protection; **DegP**	B5Z0E1_ECO5E (+1)	49 kDa
Serine endoprotease/Protection; **DegQ**	Q8X9F1_ECO57	47 kDa
Single-stranded DNA-binding protein/DNA Replication; **SsB**	SSB_ECO57	19 kDa
Soluble cytochrome, electron transport/Energy Metabolism; **CybC**	C562_ECO57 (+4)	14 kDa
Spermidine/putrescine import ATP-binding protein/Transport; **PotA**	POTA_ECO57	43 kDa
Stringent starvation protein A, stationary phase induced acid tolerance/Global Regulator; **SspA**	SSPA_ECO57	24 kDa
Stringent starvation protein B, ClpXP protease specificity enhancer/Global Regulator; **SspB**	SSPB_ECO57	18 kDa
Succinate dehydrogenase flavoprotein subunit/Energy Metabolism; **SdhA**	DHSA_ECO57	64 kDa
Tat-linked quality control protein/DNAse activity; **TatD**	Q8X8J6_ECO57	30 kDa
Thiosulfate sulfurtransferase/Anaerobic Respiration, Energy Metabolism; **GlpE**	C8TJL4_ECO26 (+2)	12 kDa
Threonine deaminase (Dehydratase)/Amino acid Biosynthesis; **IlvA**	Q8X467_ECO57	56 kDa
Transcription termination/antitermination protein/Modulates DNA Transcription; **NusG**	NUSG_ECO57	21 kDa
Transcriptional regulatory protein/Envelope Stress Response Protein/Downregulates LEE; **CpxR**	CPXR_ECO57	26 kDa
Transketolase 1, thiamin-binding/Non-oxidative Metabloism; **TktA**	C8TGU9_ECO26	72 kDa
Transketolase 2 isozyme, stationary phase induced/Non-oxidative Metabolism; **TktB**	Q8XBF1_ECO57 (+6)	73 kDa
Translation initiation factor IF-2/Protein Translation; **InfB**	IF2_ECO57	97 kDa
Trigger factor/Cell division; **TiG**	TIG_ECO57 (+1)	48 kDa
Tryptophanase/Tryptophan Metabolism, Indole Production; **TnaA**	TNAA_ECO57	53 kDa
Type II secretion pathway related protein/Transport; **EtpE**	O82884_ECO57	56 kDa
Tyrosine-protein kinase/Downregulates colanic acid production; **WzC**	WZC_ECO57	79 kDa
Uncharacterized protein/Hypothetical; **ECs1547**	Q8X3G9_ECO57 (+1)	17 kDa
Uncharacterized protein/Hypothetical; **ECs2891**	Q8X7H8_ECO57	14 kDa
Uncharacterized protein/Hypothetical; **ECs2991**	Q8X2Z1_ECO57	10 kDa
Putative oxidative stress defense protein/ Oxidative Stress; **YggE**	YGGE_ECO57	27 kDa
Uncharacterized protein/Membrane Protein; **YqjD**	YQJD_ECO57	11 kDa
UPF0042 nucleotide-binding protein/putative ATPase; **YhbJ**	YHBJ_ECO57	32 kDa
UPF0092 membrane protein, translocase/Transport; **YajC**	YAJC_ECO57	12 kDa
UPF0337 protein, putative stress response protein/Osmotic Shock; **YjbJ**	YJBJ_ECO57	8 kDa
UPF0352 protein/Uncharacterized; **YejL**	YEJL_ECO57	8 kDa
Uridylate kinase/Nucleotide Interconversion; **PyrH**	PYRH_ECO57	26 kDa
Valine--tRNA ligase/Amino acyl tRNA Biosynthesis; **ValS**	SYV_ECO57	108 kDa

^1^kDa, Kilodalton.

As observed with the Bottom-up proteomics results, none of the well-established O157 virulence factors were identified in either media after 48 h of anaerobic incubation (Additional file 2: Table S2). Specifically, the 155 up-regulated (Table 4), uRF-O157 proteins could be functionally associated with osmotic adaptation (MdoG, CreC, OsmE, YjbJ), oxidative stress pathway (KatG, DmsA_ynfE, DmsA_ynfF, YggE), heat shock response (HdlD, GrpE), carbon starvation response (SspA, SspB), anaerobic respiration (HybC, ErpA, GlpE), pH adaptation/acid resistance (SpeA, SpeB, Mrp), energy metabolism: degradation of carbon compounds (GalT, BglX, EbgA, MtlD), glycolysis/ gluconeogenesis (GpmA, SucB, FdhO, FbaB, GloB, NuoC, AceF, PoxB, SdhA), amino acid metabolism (GcvT, GcvP, HslV, IlvE, GlnA, TnaA), nitrogen and glycerolipid metabolism (GlpK), DNA degradation (RecB), biosynthetic pathways: fatty acid (FabG, AccD, AccB), amino acids (DapB, ArgH, AsD, IlvA), nucleotides (PyrI, PyrC, PurH, GlmU, CmK, GuaA, GuaB, GmK,), cellulose (BcsC), cofactors/carriers (MoaC, GshB), vitamins (RibB, PdxJ), chaperones (fimbrial usher protein,), transport (HmuV, FadL, PlaP, MacB, OppA, NikA, SecD, ManZ, PotA, YajC, EtpE), storage (BfR, FtnA), multi-drug efflux systems (AcrA, MdtC), tellurite resistance (TerC), serine proteases (DegP, DegQ, EspP), outer membrane proteins/porins/channel (AsmA, LptE, Lpp, NagA, SlyB, OmpA, BamA, BamD, TolC, OmpW, ElaB, YbjP, LppC, YqjD), chemotaxis (Trg), adherence (AidA-like, BssR, CsgG, CsgB, LpoB/YcfM, EspP), and cell division/DNA replication (HrpB, ZapB, MukB, ParE, Ssb, Tig) (Table 4; Additional file 2: Table S2).

## Discussion

This study provides a snapshot of various proteins expressed by O157 in unfiltered, rumen fluid through a comparative analysis of the O157 proteomic-profile in different media, growth conditions and incubation times. Interestingly, none of the reported (LEE, Shiga toxins) O157 virulence proteins were detected, under all conditions, in any media tested. Overall, fewer O157 proteins were detected in more nutritionally complex RF-preparations versus LB and among these, differences were observed based on availability of oxygen, nutrients and incubation time. Also, the O157-proteome in the RF-preparations included more proteins with diverse functions at 48 h than after 14 days of incubation. In fact, proteins associated with adherence, cell division and growth were identified only at 48 h. However, under all conditions, a selective expression of proteins with a role in cell structure, transport, metabolism, chemotaxis, motility, resistance, stress and regulation was observed in RF-preparations , many of which were up-regulated in the unfiltered rumen fluid. The O157 growth patterns and proteome expressed in the rumen fluid is suggestive of an adapting O157, expending minimal energy, preparing for survival and downstream intestinal colonization.

Since adult cattle are often fed a maintenance diet with less protein until ready for feedlots, we decided to analyze O157 growth dynamics in rumen fluid derived from animals on this diet. Rumen fluid from cattle fed a diet low in protein usually has a pH ranging from 6.2-6.8, and VFA concentrations at, 60-70% acetic acid, 15-20% propionic acid, 5-15% butyric acid [28-31]. The rumen fluid VFA and pH values were within the limits described for this diet for both animals used in this study (Tables 1 and 2; 26–29). Irrespective of incubation times (14 days versus 48 h), O157 exhibited very distinctive growth patterns in RF-preparations compared to LB. O157 cultures in dRF, fRF and uRF were consistently at lower optical densities than LB, under both aerobic and anaerobic conditions. The anaerobic RF-preparation cultures never reached an OD_600_ ≅ 1.0 and the viable O157 recovered were at substantially lower counts when compared to LB. The low OD readings and viable counts recovered from RF-preparation grown cultures may have been due to inhibitory factors and /or limited nutrients in dRF, fRF, uRF, not seen in LB, having a bacteriostatic (aerobic) or bactericidal (anaerobic) effect on O157 and reflective of O157 growth in a stressful environment [11,32-36]. Using LB media for estimating viable counts may have helped recover the stressed bacteria [35]. Similar recovery of viable bacteria despite low OD reading has been reported among bacteria exposed to antimicrobial stress [36], and limited growth has been associated with bacteria entering into a stressed/starved state or stationary phase [35-37].

Overall, fewer O157 proteins were detected in RF-preparation cultures compared to LB, especially under anaerobic conditions. Irrespective of the media used to culture O157, its anaerobic proteome was functionally associated only with cell structure, transport, metabolism, chemotaxis, motility, resistance, stress-related and regulation, and not O157 virulence. Previous reports have demonstrated that O157 virulence genes, especially the Shiga toxin and LEE–encoded genes, are down-regulated in LB compared to minimal media [38-40]. In addition, presence of trace amounts of glucose has also been shown to down-regulate LEE expression due to catabolite repression and/or acidic pH [38-40]. Hence, the lack of virulence gene expression in LB in this study conforms to those findings. Experiments with acid-stressed, starved bacteria have shown that these are likely to be more virulent only on recovery, and over time [35]. Even in minimal media that usually supports O157 virulence gene expression, several of these are suppressed as cultures reach the stationary phase [41]. Butyrate, a key environmental cue in LEE gene expression was limited in the RF used in this study, which may have also caused the LEE suppression [9]. Conditioned media from unrelated cultures have been shown to suppress Shiga toxin gene expression while maintaining O157 growth or suppressing growth itself [33,35,42]. In fact, experimental studies have shown that it is easier to displace O157 in unfiltered rumen fluid versus autoclaved rumen fluid, by addition of “nonfermentable” sugars in the presence of the ruminal microflora [11]. Thus, the absence of O157 virulence gene expression in RF-preparations may be reflective of the stressful growth environment, suppression due to nutrient limitations, lack of inducers, oxygen deprivation, pH fluctuations and inhibitory metabolites released by resident microbiota.

Previous studies have suggested development of acid resistance by Shiga-toxin producing *E. coli* (STEC) in the rumen as a means for better STEC survival through the ‘stomach-like’ acidic bovine abomasum [43,44] and have prescribed a role for glutamate-dependent acid resistance system (Gad system) and the tryptophanase (*tnaA*) enzyme toward this end [45]. Hughes *et al.*, recently demonstrated that O157 LEE expression is down-regulated while the Gad system is up-regulated in the rumen of cattle [46]. This observation made in animals being fed a grain diet, having a ruminal pH of 5.93, derived a role for the *SdiA* gene in sensing the acylhomoserine lactone (AHL) signals in the rumen fluid and affecting differential expression of these genes. AHLs formed by ruminal resident flora, are effective only under highly acidic pH and hydrolyze at neutral-alkaline pH [46,47]. Similarly, the Gad system that relies on the decarboxylation (*gadA/B*) of glutamate via proton consumption to increase cytoplasmic alkalinity is active at pH 4–4.6 [48]. However, other degradative amino acid decarboxylase and acid-resistance systems are activated in response to low pH (5.2 to 6.9), fermentative-anaerobic growth and stationary phase growth [48,49] and used more often than the Gad system to counter the deleterious effects of protons. We observed one such system, the arginine-dependent acid resistance system (Arg system) to be up-regulated in the RF-preparations after 48 h of anaerobic growth. Since the pH of the RF-preparations used in this study did not reach extreme acidic levels, the Gad system may not have been induced. In the Arg system, decarboxylation (*speA*) of arginine via proton consumption resulting in the formation of agmatine stabilizes the cytoplasmic pH. Agmatine is either exported via the arginine-agmatine antiporter (*aidC*) or converted (*speB*) to putresceine as part of the polyamine biosynthetic pathway.

Considering that O157 is exposed to heat-shock, starvation and stationary-phase-like growth in the rumen, it is possible that these factors enhance acid-tolerance in the bacteria through other mechanisms such as outer membrane changes and synthesis of proton transport-related protective proteins, as well [49,50]. Several stress (acid, low oxygen, osmolites, stationary phase)-responsive genes were expressed by O157 in this study, and included genes associated with the metabolism of arginine (*speA*, *speB*), lysine (*lysU*), formate (*hyC*), tryptophan (*tnaA*) and maltoporin (*lamB*), catalase (*katG*), DNA polymerase-1 (*polA*) and AidA-1 adhesin-like protein (*aidA*) [49-51]. Flagellar genes are differentially expressed under varying acid-stress conditions [51-53], and in our study, these genes were up-regulated in dRF and fRF but not uRF, suggesting less pH variation in the course of growth in uRF and limiting the role of flagella to motility alone. Stressed bacteria have been shown to be more adherent [35,40,53]; proteins associated with adherence (AidA-1 adhesin-like) and biofilm formation (BssR, CsgG, CsgB) were identified after 48 h incubation and not after longer incubation periods. Interestingly, several ‘resistance’ related proteins were up-regulated in RF-preparations, a subset of which (tellurite resistance, serine protease) have also been shown to contribute towards O157 adherence [54,55]. This suggests that adherence may be critical during the initial phase of O157 colonization and although LEE is suppressed, the bacteria rely on other mechanisms to adhere or form biofilms in the rumen. It has been observed that bacteria and protozoa in the rumen tend to adhere to the fibrous mat layers comprising of plant material to remain in the rumen and assist in the digestion of insoluble feed materials [56]. While this may not be in the case of O157, initial adherence to or biofilm formation on available surfaces may give the bacteria time to adapt and survive the rumen environment [34]. It appears that much of the adaptive changes are initiated early in colonization as reflected in more stress-induced, structural integrity-related outer membrane proteins (AsmA, LptE, Lpp, NagA, SlyB, OmpA, BamA, BamD, TolC, OmpW, ElaB, YbjP, LppC, YqjD), and cell division and growth proteins, being expressed at 48 h. This supports the observation that O157 is maintaining slow growth in the RF-preparations as well.

## Conclusion

Bottom-up proteomics provided a broad picture of differences in O157 protein expression after extended incubation in various media tested. Quantitative proteomics (iTRAQ)-based analysis of the O157 anaerobic proteome expressed in uRF with all normal rumen flora was performed to more closely determine O157 protein expression in the bovine rumen. The cumulative results of all RF-preparation analysis suggested that rumen specific protein expression enables O157 to adapt to this hostile environment and successfully transit to its colonization sites in the bovine GIT. To further verify our conclusions, we are evaluating the O157 proteomic-profile as expressed *in vivo* in a rumen-fistulated cow, and confirming the role of a subset of these ‘adaptive’ proteins in O157 survival.

## Competing interests

The authors declare no competing financial interests.

## Authors’ contributions

ITK was the project leader and designed, coordinated, conducted experiments, analyzed results, interpreted data and drafted the manuscript. TBS assisted in design of experiments, VFA analysis, interpreted results and contributed to the final draft of the manuscript. JDL conducted iTRAQ proteomics, verified data generated and contributed to the final draft of the manuscript. All authors read and approved the final manuscript.

## Supplementary Material

Additional file 1: Table S1 Bottom-up Proteomics Dataset. Click here for file

Additional file 2: Table S2 iTRAQ Proteomics Dataset. Click here for file
